# Impact of Artificial Rearing Systems on the Developmental and Reproductive Fitness of the Predatory Bug, *Orius laevigatus*

**DOI:** 10.1673/031.010.10401

**Published:** 2010-07-13

**Authors:** Maarten Bonte, Patrick De Clercq

**Affiliations:** Laboratory of Agrozoology, Department of Crop Protection, Ghent University, Coupure Links 653, B-9000 Ghent, Belgium

**Keywords:** biological control, diet, factitious prey, oviposition substrate

## Abstract

This study investigated the effect of several substrate types and moisture sources on the developmental and reproductive fitness of the zoophytophagous predator *Orius laevigatus* (Fieber) (Hemiptera: Anthocoridae) when fed a factitious prey (i.e. unnatural prey) *Ephestia kuehniella* (Zeller) eggs, or a meridic artificial diet based on hen's egg yolk. *O. laevigatus* is known to feed on plants as an alternative food source and to oviposit in plants. *E. kuehniella* eggs were superior to the artificial diet. Supplementary feeding on plant materials did not compensate for the nutritional shortcomings of the artificial diet. Survival rates showed that oviposition substrates such as bean pods or lipophilic surfaces such as wax paper and plastic were more suitable for rearing *O. laevigatus* than household paper. The use of green bean pods as a plant substrate did not have a beneficial effect on *O. laevigatus.* The results indicated that *O. laevigatus* can successfully complete its nymphal development and realize its full reproductive potential in the absence of plant material. However, plant materials would still be required for oviposition, unless a reliable and cost-effective artificial oviposition substrate were made available. The omission of plant materials from the rearing procedures may reduce production cost of this species and other heteropteran predators.

## Introduction

In order to stimulate the use of augmentative biological control, increasing effort is being put into the reduction of production costs. This involves developing economically viable and nutritionally adequate alternative foods or artificial diets (Cohen 2004; De Clercq 2008) and, for some insects, designing artificial oviposition substrates (Castañe and Zalom 1993; Constant et al. 1996). Natural rearing systems for many predators and parasitoids are typically tritrophic: the predator is reared on its prey or host, which is maintained on its own host plant. These rearing systems have several downsides. They are often expensive (due to costs related to space and labor needed for plant production), problems with discontinuity might occur for one of the trophic levels, and plant material must be free of harmful pesticide residues. Predatory heteropterans use plants or plant parts for moisture, supplementary nutrients, and oviposition substrates. The implementation of artificial diets and substrates may help rationalize production of these predators, as it would allow the omission of plant materials from the rearing system. In turn, this would reduce the need for large surfaces of greenhouses for growing host plants, leading to a drastic reduction of costs.

The anthocorid predator *Orius laevigatus* (Fieber) (Hemiptera: Anthocoridae) attacks several small arthropod crop pests but is mainly used for the augmentative biological control of the western flower thrips, *Frankliniella occidentalis* (Chambers et al. 1993; Bosco et al. 2008). *Orius* bugs are known to be facultatively phytophagous (Coll 1996; Lattin 1999). *O. insidiosus* feeds on xylem and mesophyll contents, allowing the bug to ingest water and small amounts of sugars, starches, and amino acids from the plant (Armer et al. 1998). The presence of amylase in the bug indicated that it can digest the starch from plants and thus benefit from plant feeding when prey is scarce (Zeng and Cohen 2000). Lundgren et al. (2008) showed that neonate *O. insidiosus* was able to use plant tissues for nutrition in its early developmental stages and that the bugs not only feed on xylem but also on the nutritious phloem, allowing them to survive solely on plant materials for several days. *O. laevigatus* also can complete its development on certain plant materials, such as fresh sweet pepper pollen (Vacante et al. 1997). These findings suggest that feeding on plant materials is ecologically relevant for *Orius* bugs.

Omission of plant materials in completely artificial rearing systems may negatively influence the developmental and reproductive fitness of the insect. In addition to direct feeding on plants, *Orius* bugs also use plants as substrates for egg laying, inserting their eggs into the tissue using an ovipositor. *O. insidiosus* females insert their eggs into the thinnest external plant tissue, which enhances the survival of their offspring. This suggests the importance of plant feeding during the early developmental stages of the predator (Lundgren et al. 2008). Further, since *Orius* bugs are thigmotactic insects that are often found in flowers (Chambers et al. 1993; Lattin 1999) the lack of plants as natural shelter might have an impact on their behavior. Lack of suitable hiding places could lead to greater stress, resulting in energy loss and affecting the overall fitness of the insect in its rearing environment.

This study tested the effect of several substrate types and water sources on the developmental and reproductive parameters of *O. laevigatus* fed on two different types of diet (a factitious or unnatural prey and an artificial diet).

## Materials and Methods

The stock colony of *O. laevigatus* was initiated with insects acquired from Biobest NV. (www.biobest.be) and held at 23 ± 1° C, 65 ± 5% relative humidity, and 16:8 light:dark. The insects were reared on sharp pepper plants (*Capsicum annuum* L. cv. ‘Cayenne Long Slim’) and were offered *Ephestia kuehniella* (Zeller) eggs as food.

Two experiments were performed to assess the effect of moisture source and living substrate on the development and reproduction of *O. laevigatus* fed on two diets: (1) frozen eggs of the Mediterranean flour moth *E. kuehniella* (supplied by Koppert BV, Berkel en Rodenrijs, The Netherlands) or (2) a meridic artificial diet developed for *O. laevigatus* by Arijs and De Clercq (2002). The artificial diet was composed of 3 g casein, 2.5 g casein hydrolysate, 2 g soy hydrolysate, 3 g lactalbumin, 30 g fresh hen's egg yolk, 3 g soy oil, 1 g peanut oil, 1 g dextrose, 0.5 g Wesson's salt mix, 53.9 g water, 0.06 g of a vitamin mix based on the vitamin composition of bovine liver (weight percentages: 25.4% nicotinic acid, 4.9% riboflavin, 0.5% thiamine, 1.5% vitamin B6, 12.4% Capantothenate, 1% folic acid, 0.1% biotin and 54.2% vitamin C), and 1 mg vitamin E. The artificial diet was prepared on a weekly basis and kept in a refrigerator at 4° C. Both the diet and water were encapsulated into small hemispherical domes (70 µl) consisting of Parafilm M using an encapsulation device (ARS, Gainesville, Florida). Stretching the Parafilm M before encapsulation facilitated stylet penetration by early instars of the insect. The domes were sealed using transparent tape (Scotch 3M Packaging Super Tape, 3M, www.3m.com). All foods were supplied *ad libitum*; artificial food was replaced daily, whereas the *E. kuehniella* eggs were replenished every other day. In both experiments, predators were caged in individual plastic containers (5 cm diameter, 2 cm high) with two ventilation holes screened with fine-mesh nylon gauze.

In the first series of tests, four different water sources were examined. In the first treatment, a sharp pepper seedling was provided with its roots immersed in water. In the second treatment, water was provided by a Parafilm dome filled with tap water and frozen moist bee pollen (Koppert BV, www.koppert.com) was supplied as a plant-derived food. In the third treatment, a Parafilm dome filled with a 5% sucrose solution was offered; whereas, in the fourth, only a Parafilm dome with tap water was offered. The Parafilm domes were made using an encapsulation device as described above. As some of the water sources used also provided nutrients, “water source” is used as an operational term and is not meant to be physiologically defining.

Four different types of substrates were tested in a second experiment. The treatments were: no extra substrate, wax paper, household paper, and a pod of flat green bean (*Phaseolus vulgaris* L.). The flat green bean pod was cut between two seeds into 2–3 cm pieces to prevent nymphs from hiding inside the bean. Wax paper consisted of kraft paper with paraffin impregnations. The household paper used was absorbent 2-ply paper towel (Tork Premium Kitchen Roll, SCA, www.scatork.com). The wax paper and household paper were cut into squares (5 × 5 cm) and placed on the bottom of a container. Each container was provided with either diet and a Parafilm dome filled with tap water.

In each treatment of each experiment, 40 first instars (< 24 h old) were placed individually in the experimental containers and allowed to develop to the adult stage. Survival and development of nymphs were monitored daily, and newly emerged adults were sexed and weighed using a Sartorius Genius ME215P balance (Sartorius, www.sartorius.com). Adults were randomly mated and given the same diet they received as nymphs. The predator's reproductive capacity was predicted by assessing ovarian development according to the method described by Bonte and De Clercq (2008). For this purpose, 8-day-old females were dissected, and oocytes (follicles) in ovaries and oviducts were counted.

### Statistical analyses

Survival rates were compared by means of a logistic regression. This regression is a generalized linear model using a logit (log odds) link and a binomial error function. Each test consists of a regression coefficient that is calculated and tested for being significantly different from zero, for which p-values are presented (McCullagh and Neider 1989). p-values smaller than or equal to 0.05 are considered significant (StataCorp 2005). The parameters of developmental time and adult weight were analyzed using a one-way analysis of variance (ANOVA), and means were separated using a suitable post hoc test; Tukey and Tamhane tests were chosen in cases of equal or unequal variances, respectively (Tamhane 1979; SPSS Inc. 2006). Homogeneity of variance was tested by Levene statistics. A Kruskal-Wallis one-way ANOVA with Bonferroni correction was performed to determine differences in oocyte counts, and means were separated using a Mann-Whitney U test. Means were subjected to a two-way ANOVA when *O. laevigatus* was exposed to different water sources or substrate types to assess whether diet had an effect on developmental time, weight, or oocyte counts (SPSS Inc. 2006).

**Table 1.**
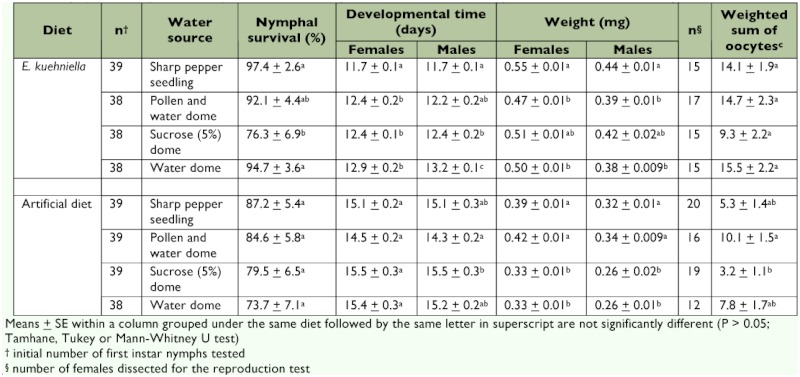
Nymphal development and reproduction of *O. laevigatus* on different substrate types when fed on *E. kuehniella* eggs or an artificial diet.

## Results

### Effect of moisture source

Nymphal survival of *O. laevigatus* that were fed eggs of *E. kuehniella* was better for those offered a sharp pepper seedling (97.4%) or a water dome (94.7%) as a moisture source than for those given a sucrose solution (76.3%) (Table 1). The moisture source had no influence on the survival of nymphs fed artificial diet. The developmental time of both females and males fed *E. kuehniella* eggs and offered a sharp pepper seedling (11.7 days for both sexes) was significantly shorter than with the other treatments, except when pollen or water (12.4 and 12.2 days, respectively) were supplied (Table 2). The moisture source had only a marginally significant impact on the developmental time of females fed artificial diet but did affect the developmental time of males. Males fed artificial diet and given pollen and water developed faster (13.4 days) than those given a sucrose solution (15.5 days). Weight of both sexes fed *E. kuehniella* eggs was significantly higher for *O. laevigatus* supplied with a sharp pepper seedling than for those supplied with water only or with pollen and water. For males and females fed artificial diet, insects offered a sharp pepper seedling (0.32 and 0.39 mg, respectively) or pollen and water (0.34 and 0.42 mg, respectively) were significantly heavier than those offered a sucrose solution (0.26 and 0.33 mg, respectively) or those given water only (0.26 and 0.33 mg, respectively). Moisture source had no effect on the number of oocytes counted in females fed eggs of *E. kuehniella.* Oocyte counts of females fed artificial diet supplemented with pollen and water were significantly higher than of those supplied with 5% sucrose solution. Female adults kept on a sharp pepper seedling during the 8 day experimental period deposited 16.0 ± 3.1 eggs (mean ± SE) and 3.6 ± 1.9 eggs in the plant when fed on *E. kuehniella* eggs and artificial diet, respectively. Two-way ANOVA showed significant interactions between food and moisture source for the parameters of developmental time and weight but not for oocyte counts (Table 3). For all tested parameters, eggs of *E. kuehniella* as a food scored better than the artificial diet. Moisture source significantly affected all tested parameters.

### Effect of substrate

Survival of nymphs fed *E. kuehniella* eggs and reared on a piece of bean (92.4%) was significantly better than that of those offered the same food but household paper as a substrate (74.4%) (Table 4). On the artificial diet, development of nymphs reared on household paper (57.5%) was significantly less successful than of those given no extra substrate at all (84.6%). The presence of wax paper and household paper substrates had a positive influence on the developmental time of both males and females fed *E. kuehniella* eggs but not of those fed artificial diet (Table 5). Both females and males fed *E. kuehniella* had shorter developmental times when reared on wax paper (12.1 and 12.2 days, respectively) or household paper (12.4 and 12.1 days, respectively) than those reared on a bean pod (13.6 and 13.7 days, respectively) or without an extra substrate (13.5 and 13.3 days, respectively). For either food, substrate had no effect on adult weight of males, but did influence adult weight of females. Females fed *E. kuehniella* were heavier when reared on wax paper (0.52 mg) than those reared on household paper (0.45 mg) or without an extra substrate (0.47 mg). On the other hand, females fed artificial diet and reared in containers without a substrate had higher weights (0.35 mg) than those reared on a bean pod (0.32 mg). The type of substrate did not influence the number of oocytes counted in females whether fed eggs of *E. kuehniella* or artificial diet. Two-way ANOVA indicated that the diet x substrate interaction was significant for developmental time and adult weight but not for oocyte counts (Table 6). For all tested parameters, *E. kuehniella* eggs were superior to the artificial diet as a food source. Substrate as a main effect significantly influenced the parameters of developmental time and adult weight but not the oocyte counts.

**Table 2.**
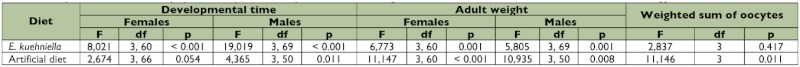
One-way ANOVA results for nymphal development and reproduction of *O. laevigatus* on different water sources when fed on *E. kuehniella* eggs or an artificial diet

**Table 3.**
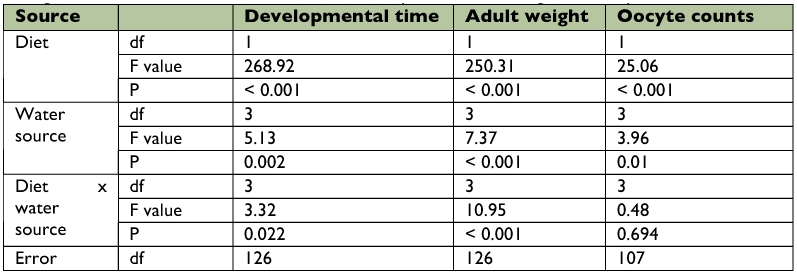
Two-way ANOVA results indicating the effect of diet and water source on developmental time, weight and oocyte counts.

**Table 4.**
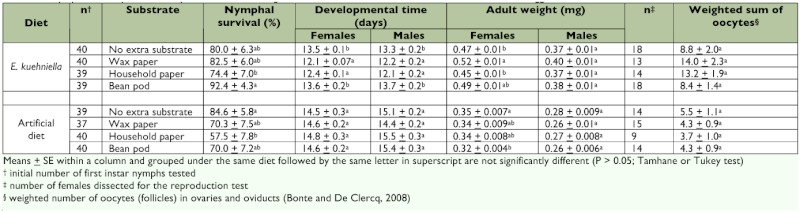
Nymphal development and reproduction of *O. laevigatus* on different water sources when fed on *E. kuehniella* eggs or an artificial diet.

**Table 5.**
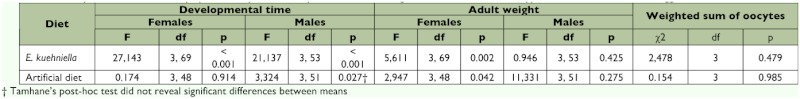
One-way ANOVA results for nymphal development and reproduction of *O. laevigatus* on different substrate types when fed on *E. kuehniella* eggs or an artificial diet.

## Discussion

The experimental setup described here did not allow for fully separating the effects of diet and moisture source and of diet and living substrate. For instance, several of the moisture sources used also provided nutrients, whereas the diets themselves also serve as a source of water as they are metabolically processed to yield water. As a result, analysis of variance indicated a strong statistical interaction between diet and moisture source and diet and living substrate for the parameters of developmental time and adult weight, implying that none of the factors alone could sufficiently explain the observed differences.

The developmental and reproductive performance of *O. laevigatus* reared on eggs of *E. kuehniella* was superior to that of those reared on a meridic artificial diet, which is in accordance with earlier findings (Bonte and De Clercq 2008). In addition to differences in nutritional value, better performance of *O. laevigatus* when fed on *E. kuehniella* eggs rather than on artificial diet domes may be related to size differences among these foods: the volume of a diet dome was over 2,000 times that of an individual flour moth egg. A solitary feeding heteropteran predator may be faced with a lower efficiency of its extra-oral digestion when feeding on larger volume prey or artificial diet packages (Cohen and Tang 1997; Ferkovich et al. 2007).

**Table 6.**
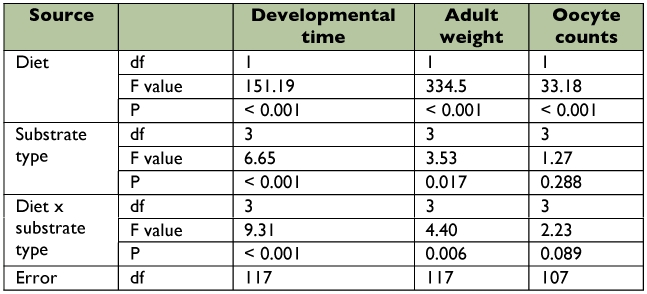
Two-way ANOVA results indicating the effect of diet and substrate type on developmental time, adult weight and oocyte counts.

Overall, sharp pepper seedlings yielded faster development and greater adult weights than the other moisture sources when the predator was fed on *E. kuehniella* eggs. In addition to extracting water and supplemental nutrients from the plant (Armer et al. 1998; Lundgren et al. 2008), *O. laevigatus* may have benefited from living on a suitable substrate. Several studies have shown that plant material can compensate for the dietary shortcomings of nutritionally inadequate diets. Gillespie and McGregor (2000) have shown that the addition of tomato leaves to a diet of *E. kuehniella* eggs decreased nymphal mortality and shortened development of the mirid predator *Dicyphus hesperus.* Patt et al. (2003) found that the inclusion of pollen and sucrose into a diet consisting of the suboptimal factitious prey *Drosophila melanogaster* enhanced the growth of the green lacewing *Chrysoperla carnea.* De Clercq et al. (2005) found that supplementing pollen to a diet of *E. kuehniella* eggs increased egg hatch in the two-spotted ladybeetle *Adalia bipunctata.* In this study, the beneficial effect of moist bee pollen was less profound than that of seedlings when *O. laevigatus* was fed *E. kuehniella* eggs. On artificial diet, however, neither plant food was able to compensate for the nutritional deficiencies of the diet. Survival, developmental times, and oocyte counts were similar for diet-fed *O. laevigatus* offered plant foods or water, but adult weights were higher when artificial diet was supplemented with either pollen or a sharp pepper seedling. The addition of sucrose to the water dome did not benefit the overall fitness of *O. laevigatus.* On the contrary, when *O. laevigatus* were provided with *E. kuehniella* eggs, survival of nymphs offered a sucrose solution was lower than that of those offered water only.

Survival rate of nymphs was lowest on household paper with both diets. This indicated that green bean pods and lipophilic substrates such as wax paper and polystyrene plastic were superior living substrates for *O. laevigatus*. On the other hand, the use of artificial substrates such as wax paper and household paper in combination with eggs of *E. kuehniella* yielded shorter developmental times than when a green bean pod or no extra substrate were provided. In the substrate experiment, *O. laevigatus* did not appear to have benefited from the bean pod as a supplementary plant food. This might be related to the fact that the bean pods were replaced only twice a week, leading them to desiccate. Sláma and Williams (1965) found that there were risks involved in using paper in rearing systems containing substances with juvenile hormone activity. However, no such abnormalities were ever observed at our facilities in cultures of different insects where this paper is routinely used. The findings suggest that providing no extra substrate to a rearing container is equally effective but evidently cheaper than when extra materials are to be added as living substrates for *O. laevigatus.* However, these experiments were performed using isolated nymphs; whereas, in communal cultures, extra substrates may be needed to prevent cannibalism, particularly when suboptimal alternative foods are given (Grenier and De Clercq 2003). The shape of substrates may play an important role in mediating interactions, especially cannibalism, among insects. For instance, seedlings provide a more complex spatial environment than artificial substrates like Parafilm domes.

Coll (1996) pointed out the omnivorous nature of *O. insidiosus* and showed that besides providing oviposition sites, plants may also serve as alternative foods for the predator. However, this study shows that *O. laevigatus* can successfully complete its nymphal development and realize its full reproductive potential without plant materials. In a rearing environment, plants may still be needed as oviposition substrates, unless an effective and inexpensive oviposition substrate can be found. Castañe and Zalom (1993) developed an artificial oviposition substrate for *O. insidiosus* by coating a drop of gelatin with a thin paraffin wax layer. Females successfully oviposited in this artificial substrate and the eggs hatched successfully but eggs were never found when the paraffin layer was thicker than 0.045 mm. For the mirid bug, *Macrolophus caliginosus,* which also inserts its eggs into plant tissue, a moist dental cotton roll wrapped in stretched Parafilm allowed egg laying and embryonic development (Constant et al. 1996). In this study *O. laevigatus* females deposited eggs during the 8-day experimental period in the sharp pepper seedling but not into the Parafilm domes containing or into the bean pods. In the experiments on moisture sources, the use of sharp pepper seedlings may have affected oocyte counts used as a measure of fecundity. As some females had oviposited into the seedlings, this may have influenced the number of oocytes remaining in the female at dissection. In contrast, oviposition into pepper seedlings before day 8 in adult life was not observed in an earlier study (Bonte and De Clercq 2008), which may be related to differences in age of the seedlings used. Although bean pods are proposed by van Lenteren et al. (2003) as oviposition substrates to assess fecundity of *Orius* spp. in the framework of quality control, no eggs were found in the bean pods used in this experiment. The desiccated state of the bean cuttings may have deterred females from depositing eggs into them. Alternatively, the bean pods, originating from a commercial source, may have been contaminated with pesticide residues. In contrast to the findings from this study, Shapiro and Ferkovich (2006) found that *O. insidiosus* females deposited eggs in water-filled Parafilm domes. However, the domes used in the recent tests were about seven times smaller than those used by Shapiro and Ferkovich (2006), and the thickness of the Parafilm layer may have been different, potentially affecting the oviposition behavior of *O. laevigatus.* Further studies are needed to develop artificial oviposition substrates for predatory heteropterans. In the commercial production of these economically important natural enemies, the availability of high quality artificial oviposition substrates may eliminate the need to maintain large surfaces of greenhouses for plant growing as well as the costs associated with this activity.
